# Aphasic mild cognitive impairment in prodromal dementia with Lewy bodies

**DOI:** 10.3389/fneur.2023.1128566

**Published:** 2023-04-03

**Authors:** Hiroyuki Watanabe, Sakura Hikida, Manabu Ikeda, Etsuro Mori

**Affiliations:** ^1^Department of Behavioral Neurology and Neuropsychiatry, Osaka University United Graduate School of Child Development, Suita, Japan; ^2^Department of Psychiatry, Osaka University Graduate School of Medicine, Suita, Japan; ^3^Brain Function Center, Nippon Life Hospital, Osaka, Japan

**Keywords:** Lewy body disease, Alzheimer's disease, mild cognitive impairment, primary progressive aphasia, cholinesterase inhibitor

## Abstract

**Introduction:**

This study aimed to determine the characteristics of aphasic mild cognitive impairment (aphasic MCI), which is characterized by a progressive and relatively prominent language impairment compared with other cognitive impairments, in the prodromal phase of dementia with Lewy bodies (DLB).

**Methods:**

Of the 26 consecutive patients with aphasic MCI who had been prospectively recruited at our hospital, 8 patients were diagnosed with prodromal DLB and underwent language, neurological, neuropsychological, and neuroimaging (*N*-isopropyl-p-[^123^I] iodoamphetamine single-photon emission computed tomography; IMP-SPECT) testing. Three of these patients also underwent cholinesterase inhibitor therapy with donepezil.

**Results:**

In our aphasic MCI cohort, the clinical diagnosis of probable prodromal DLB accounted for more than 30% of cases; therefore, the presence of language impairment in prodromal DLB was not very uncommon. Five patients were diagnosed with progressive anomic aphasia and three with logopenic progressive aphasia. Anomic aphasia was characterized by apparent anomia but relatively preserved repetition and comprehension ability and logopenic progressive aphasia by anomia, phonemic paraphasia, and impaired repetition. IMP-SPECT revealed hypoperfusion of the temporal and parietal lobes in the left hemisphere in all but one patient. All patients who underwent cholinesterase inhibitor therapy with donepezil showed improvement in general cognitive function, including language function.

**Discussion:**

The clinical and imaging features of aphasic MCI in prodromal DLB are similar to those observed in Alzheimer's disease. Progressive fluent aphasia, such as progressive anomic aphasia and logopenic progressive aphasia, is one of the clinical presentations in prodromal state of DLB. Our findings provide further insight into the clinical spectrum of prodromal DLB and may contribute to the development of medication for progressive aphasia caused by cholinergic insufficiency.

## 1. Introduction

Dementia with Lewy bodies (DLB) is the second most common type of neurodegenerative dementia (1). Various clinical symptoms are observed in patients in the prodromal phase (pre-dementia stage) of DLB. In 2020, an international DLB study group proposed three clinical presentations of prodromal DLB: (1) mild cognitive impairment with Lewy bodies (MCI-LB), (2) delirium-onset, and (3) psychiatric-onset presentations (2). The clinical features of cognitive impairments in DLB and MCI-LB include deficits in attention, executive function, and visual perception (2, 3). Conversely, language function is normally well-preserved in DLB and MCI-LB (2, 3).

Regarding language impairment in neurodegenerative disease, primary progressive aphasia (PPA) has been defined as a neurological syndrome characterized by progressive and relatively predominant language impairment (4). PPA can be categorized into three clinical syndromic variants: non-fluent/agrammatic variant of PPA (nfvPPA), semantic variant of PPA (svPPA), and logopenic variant of PPA (lvPPA) (5). Our research group recently proposed additional fluent variants: primary progressive anomic aphasia, primary progressive transcortical sensory aphasia (TCSA), and primary progressive Wernicke's aphasia (6). PPA is associated with neuropathological diseases, such as all major forms of frontotemporal lobar degeneration and Alzheimer's disease (7). However, Lewy body disease have rarely been reported in patients with PPA.

Understanding language impairment, including PPA, in MCI-LB is crucial for investigating a wide range of clinical spectrums of prodromal DLB, and provides an opportunity to develop medications for progressive aphasias. However, the clinical and imaging features of language impairment in prodromal DLB remain incompletely understood because all but three studies—specifically, one study of six patients with lvPPA (8) and two additional studies of eight patients with PPA (9) and nine patients with PPA (10), respectively—are single case reports (11–18). Previous studies have suggested that lvPPA (8, 11, 12, 14, 15, 17) or primary progressive anomic aphasia (16, 19) are mainly observed in prodromal DLB; therefore, the characteristics of language impairments in prodromal DLB might be similar to those observed in Alzheimer's disease (6, 20, 21). This study aimed to determine the characteristics of aphasic MCI, including PPA, in prodromal DLB (aphasic MCI-LB). Here, we use the term “aphasic MCI” to characterize a progressive and relatively prominent language impairment compared with other cognitive impairments. The term “aphasic MCI-LB” is used to characterize aphasic MCI in prodromal DLB. In addition, we use the term “progressive aphasia” to simply characterize a progressive language impairment. In other words, progressive aphasia includes both PPA and non-PPA, such as aphasic MCI-LB. In this study, the language features of lvPPA, primary progressive anomic aphasia, primary progressive TCSA, and primary progressive Wernicke's aphasia in “PPA” correspond to logopenic progressive aphasia, progressive anomic aphasia, progressive TCSA, and progressive Wernicke's aphasia in “progressive aphasia,” respectively.

## 2. Materials and methods

### 2.1. Participants

We identified consecutive patients with aphasic MCI-LB who were prospectively recruited at Nippon Life Hospital, Osaka, Japan, between September 2018 and August 2021. The inclusion criteria were as follows: (1) diagnosis of aphasic MCI and (2) diagnosis of probable MCI-LB (2). The exclusion criteria were as follows: (1) significant impairment in cognitive domains other than language; (2) history of neurological diseases other than prodromal DLB, psychiatric diseases, or hearing impairment; and (3) evidence of focal brain lesions other than atrophy on magnetic resonance imaging. All patients underwent standard neuropsychological, speech, and language assessments by an experienced speech and language therapist and clinical neuropsychologist and routine laboratory investigations. *N*-isopropyl-p-[^123^I] iodoamphetamine single-photon emission computed tomography (IMP-SPECT) were performed. The diagnostic classification was blinded to the results of the imaging analysis and was based on the results and recordings of the speech and language assessments and samples of general conversation, which were reviewed in a consensus meeting 2–4 weeks after the clinical assessments.

All patients and their caregivers provided written informed consent. This study was conducted in accordance with the principles of the Declaration of Helsinki and was approved by the Ethics Committee of Nippon Life Hospital.

### 2.2. Background neuropsychological and behavioral assessments

To determine the features of cognitive and behavioral alterations, all patients underwent the following tests: Clinical Dementia Rating (CDR) (22); Mini Mental State Examination (MMSE); Addenbrooke's Cognitive Examination-Revised (ACE-R) (23, 24); Alzheimer's Disease Assessment Scale (ADAS) (25); digit span and spatial span tests; and noise pareidolia tests (26, 27), in which visual hallucination-like illusions were evoked and measured.

The following questionnaires were administered to the patients' caregivers: the Neuropsychiatric Inventory (28) to assess hallucinations and sleep disturbances and the Cognitive Fluctuation Inventory (29) to assess fluctuations in cognition.

### 2.3. Language assessment

After bedside language assessments (30), the following in-depth evaluations were conducted: the Western Aphasia Battery (WAB) (31, 32), which served as the primary measure of general language ability; and the Test of Lexical Processing in Aphasia (TLPA) (33). The TLPA is a standardized, widely used language test for Japanese speakers, and a total of 200 items in line-drawing cards are included in the picture-naming task or the auditory word comprehension task. In the TLPA-word comprehension task, after listening to a spoken word, patients are asked to match one of 10 line-drawing cards. The TLPA-word comprehension task was used to classify the severity of single-word comprehension impairment. TLPA-word comprehension scores were categorized as follows: normal, 198–200; minimal impairment, 181–197; mild impairment: 161–180; severe impairment, <161 (6).

### 2.4. Determination of the extent of hypoperfusion

The extent of hypoperfusion on IMP-SPECT was determined by three-dimensional (3D) stereotactic surface projections (SSPs) (34), in which IMP-SPECT images were sampled at 16,000 predefined cortical locations and projected on the 3D image after realignment, spatial normalization, and non-linear warping. The voxel values of an individual's IMP-SPECT data were normalized to the whole-brain tracer uptake and compared with an age-matched normal database, yielding a 3D SSP Z-score image; the abnormalities of cerebral hypoperfusion were displayed with a Z-score map. Z-scores were calculated using the following equation: Z-score = [(control mean)–(individual value)]/(control standard deviation). We used a Z-score of 2 as the cut-off value in each voxel, and voxels with a Z-score of ≤2 were considered voxels without significantly decreased regional cerebral blood flow.

## 3. Results

### 3.1. Demographics of patients

**Figure 1:**
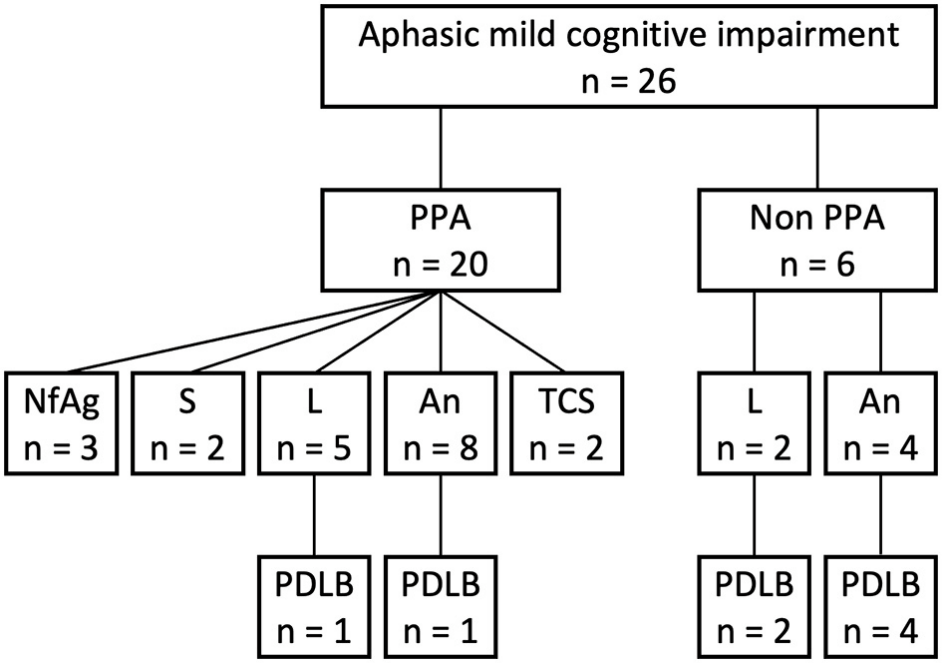
Flowchart of participant selection. An, anomic; L, logopenic; NfAg, non-fluent/agrammatic; PDLB, prodromal dementia with Lewy bodies; PPA, primary progressive aphasia; S, semantic; TCS, transcortical sensory.

Eight patients with both aphasic MCI and probable MCI-LB were enrolled in this study (Figure 1). During the study enrollment period, 26 patients were diagnosed with aphasic MCI at the Brain Function Center of Nippon Life Hospital. Of the 26 patients, 20 met the core criteria for PPA. Of the 20 patients with PPA, three met the criteria for nfvPPA, two for svPPA, five for lvPPA, and 10 for the unclassified fluent variant of PPA (primary progressive anomic aphasia, *n* = 8; primary progressive TCSA, *n* = 2). Moreover, of the 20 patients with PPA, two met the criteria for probable MCI-LB (lvPPA, *n* = 1; primary progressive anomic aphasia, *n* = 1). Of the remaining six patients who did not meet the core criteria for PPA, two had logopenic progressive aphasia, and four had progressive anomic aphasia. Thus, of the 26 patients with aphasic MCI, eight (30.8%) were finally included in the study because of a diagnosis of probable MCI-LB (logopenic: *n* = 3; anomic: *n* = 5). One patient, whose case has been published as a single case report (16), was not enrolled in the current study. For clarity, case numbers were arranged in order of the WAB-Aphasia quotient score.

Three patients were women, and all but one patient (case 5) were right-handed (Table 1). The median age of onset was 69.5 years (62–81 years) [median (range)]. Two patients experienced symptom onset before 65 years of age. All eight patients had experienced language impairment for ≤5 years; of these, the majority (5/8) had experienced language impairment for ≤3 years. The results of routine laboratory tests were unremarkable. None of the patients had a family history of neurological disease.

**Table 1:** Demographics and characteristics of patients with MCI-LB.

**Characteristics**	**Case no**.										
	**1**	**2**	**2**	**3**	**4**	**4**	**5**	**6**	**7**	**8**	**8**
		**Visit 1**	**Visit 2**		**Visit 1**	**Visit 2**				**Visit 1**	**Visit 2**
CDR total score (0–3)	0.5	0.5	0.5	0.5	0.5	0.5	0.5	0.5	0.5	0.5	**2**
Sex	M	W	W	W	M	M	W	M	M	M	M
Handedness	R	R	R	R	R	R	Both	R	R	R	R
Education	22	14	14	12	16	16	12	12	16	12	12
Duration from onset (years)	4	2	3	5	1	2	1	4	1	3	4
Age at onset (years)	62	68	68	62	75	75	81	66	72	71	71
MMSE total score (30)	28	27	**20**	27	29	28	26	**25**	**24**	**15**	**8**
ACE-R total score (100)	79	82	**63**	80	83	**72**	89	**62**	75	DNT	DNT
ADAS Recall (30)	**9**	**13**	**8**	**13**	17	**10**	24	**10**	**12**	DNT	DNT
ADAS Recognition (36)	31	27	**19**	28	35	31	35	35	33	DNT	DNT
Digit span	F5B3	F6B4	F5B3	F6B4	F6B4	F7B5	**F4B3**	F5B3	F5B3	**F3B2**	**F2B1**
Spatial span	**F4B3**	F6B6	F6B4	F6B5	F5B5	F6B5	F5B4	F5B4	**F4B4**	F6B6	**F4B2**
Noise pareidolias (32)	0	0	0	DNT	DNT	**4**	**2**	0	0	DNT	DNT
**Core clinical features of MCI-LB**											
Visual hallucinations				+			+		+		
Parkinsonism	+	+		+			+	+	+		+
Cognitive fluctuations	+			+		+	+	+	+		+
RBD	+	+		+			+	+			
**Proposed biomarkers of MCI-LB**											
Reduced DAT uptake	+	+						+	+		+
Reduced MIBG uptake	+			+		+					+
**Core criteria of PPA**											
					Fit					Fit	

ACE-R, Addenbrooke's Cognitive Examination-Revised; ADAS, Alzheimer's Disease Assessment Scale; B, backward; CDR, Clinical Dementia Rating; DAT, dopamine transporter; DNT, did not test; F, forward; M, man; MCI-LB, mild cognitive impairment with Lewy bodies; MIBG, meta-iodobenzylguanidine; MMSE, Mini-Mental State Examination; PPA, primary progressive aphasia; RBD, Rapid eye movement (REM) sleep behavior disorder; W, woman. The maximum score is noted in each row header. Boldfacing represents values that are considered abnormal.

### 3.2. Clinical features, biomarkers, and the diagnosis of MCI-LB

All patients met the criteria for probable MCI-LB (Table 1) (2). None of the patients showed significant generalized dementia (CDR score: 1–3), as measured by the CDR at visit 1. Episodic memory for daily events was relatively well preserved in all patients compared with language function. However, the majority showed scores on the ADAS word recall subtest below the lower limit of healthy individuals. Similarly, three patients (cases 6–8) showed total scores on the MMSE and/or ACE-R below the lower limit of healthy individuals. All these general cognitive tasks, including memory tasks, more or less rely on language function, and the scores are affected by aphasia, in particular word-finding difficulty. However, all but one patient (case 8) did not show a score >2 SD below the mean on the ADAS word recognition subtest, which is considered to be less affected by word-finding difficulties than the ADAS word recall subtest. These findings indicated that general cognitive function except for language function was well preserved in all but one patient (case 8). Case 8 had mild episodic memory and visuospatial impairments. However, his language impairment was more prominently severe than other cognitive impairments, and was the primary cause of impaired activities of daily living (5).

Regarding the core clinical features of MCI-LB (2), three patients (37.5%) exhibited visual hallucinations, seven patients (87.5%) had Parkinsonism, seven patients (87.5%) had fluctuating cognition, and five patients (62.5%) exhibited rapid eye movement sleep behavior disorder.

Regarding the proposed biomarkers of MCI-LB (2), three patients were assessed by dopamine transporter (DAT) SPECT imaging, and all three showed reduced DAT uptake. Two patients were assessed by ^123^I-metaiodobenzylguanidine (^123^I-MIBG) myocardial scintigraphy, and both showed decreased cardiac MIBG uptake. Additionally, two patients were assessed by both DAT-SPECT imaging and ^123^I-MIBG myocardial scintigraphy, and both showed reduced DAT uptake and decreased cardiac MIBG uptake. The remaining patient (case 5) was not assessed by DAT-SPECT imaging or ^123^I-MIBG myocardial scintigraphy but showed all core clinical features of MCI-LB (2).

At visit 1, two patients (cases 4 and 8) showed no prominent cognitive, behavioral, or psychiatric symptoms, except for progressive aphasia but reported experiencing the core clinical features of MCI-LB at visit 2.

### 3.3. Features of progressive aphasias

The language features are summarized in Table 2. At visit 1, the median WAB-Aphasia quotient score was 85.4 (74.2–93.6). All patients had fluent spontaneous speech, and there was no evidence of typical nfvPPA features (5), including dysprosody, apraxia of speech, and agrammatism. In addition, none of the patients showed typical svPPA features (5) that reflect a loss of semantic knowledge, such as poor word comprehension with an obvious loss of single-word meaning, surface dyslexia/dysgraphia, visual impairments in face and object recognition, or object misuse (4, 35–40). Three patients (cases 6–8) showed typical lvPPA features (5), including anomia, phonemic paraphasia, and impaired repetition of polysyllabic words or phrases. Thus, they were diagnosed with logopenic progressive aphasia. However, the remaining five patients (cases 1–5) did not show the typical features of nfvPPA, svPPA, or lvPPA (5). Their single-word comprehension ability was within the normal/minimal range of the TLPA-word comprehension task. Moreover, repetition of polysyllabic words or phrases formed by up to four words of 14 morae on the WAB-Repetition task (“*Nihon Koko yakyu remmei*” “Japan High School Baseball Federation”) was preserved. Thus, the severity of the repetition deficit was too subtle as the typical lvPPA feature (6, 41). In addition, none of these patients showed phonemic paraphasia. Their language features were characterized by apparent anomia, and their repetition and comprehension abilities were relatively preserved. Therefore, based on the current research findings for unclassified fluent variants of PPA (6, 42, 43), five patients (cases 1–5) were diagnosed with progressive anomic aphasia.

**Table 2:** Patients' performance in language tests.

**Characteristics**	**Case no**.										**Normative data, mean (SD)**
	**1**	**2**	**2**	**3**	**4**	**4**	**5**	**6**	**7**	**8**	
		**Visit 1**	**Visit 2**		**Visit 1**	**Visit 2**				**Visit 2**	
**WAB**											
Aphasia quotient (100)	93.6	91.4	86	91.4	85.4	82.8	84.2	81	74.2	46.4	97.7 (3.0)
Fluency (10)	9	9	9	9	8	8	8	8	8	6	10.0 (0)
Information content (10)	10	9	9	9	8	8	7	8	7	5	9.7 (0.6)
Auditory comprehension (10)	8.9	9.2	9.3	9.8	9.1	9.5	9.4	9	8.1	4	9.8 (0.1)
Repetition (10)	10	9.2	7.6	9	9.9	8.4	8.8	7.8	7	5.4	9.9 (0.3)
Naming (10)	8.9	9.3	8.1	8.9	7.7	7.5	8.9	7.7	7	2.8	9.5 (0.6)
Praxis (60)	60	59	59	60	60	60	59	59	60	DNT	59.8 (0.7)
**TLPA**											
Naming (200)	178	174	150	170	119	101	177	163	161	DNT	193.4 (5.4)
Comprehension (200)	200	200	199	199	193	155	200	192	189	DNT	199.4 (1.0)
Severity of word comprehension impairment	–	–	–	–	^*^	^***^	–	^*^	^*^	^***^	
**Types of aphasia**											
	A	A	L	A	A	T	A	L	L	W	

A, progressive anomic aphasia; DNT, did not test; L, logopenic progressive aphasia; SD, standard deviation; T, progressive transcortical sensory aphasia; TLPA, Test of Lexical Processing in Aphasia; W, progressive Wernicke's aphasia; WAB, Western Aphasia Battery. The maximum score is noted in each row header. Severity of word comprehension impairment: –, normal; ^*^, minimal; ^**^, mild; ^***^, severe. Case 8 with logopenic progressive aphasia did not undergo detailed language examinations at visit 1 because of his refusal.

At visit 2, three patients (cases 2, 4, and 8) underwent follow-up examinations after 1 year (Table 2). None of the three patients showed non-fluent features such as dysprosody, apraxia of speech, or agrammatism (5). In case 4, word comprehension impairment in the TLPA-word comprehension task drastically progressed to severe at visit 2. In contrast, the severity of the repetition deficit was mild compared to that of typical lvPPA (6, 41) because the repetition ability of polysyllabic words or phrases formed by up to four words of 14 morae on the WAB-Repetition task was preserved. Thus, the pattern of aphasia at visit 2 changed from progressive anomic aphasia to progressive TCSA, characterized by relatively preserved repetition but poor word comprehension (6). In contrast with case 4, the word comprehension ability of case 2 in the TLPA-word comprehension task was within the normal range, but the repetition ability of polysyllabic words or phrases in the WAB-Repetition task deteriorated at visit 2. Thus, the pattern of aphasia changed from progressive anomic aphasia to logopenic progressive aphasia at visit 2. In case 8, general language function, including word comprehension ability for familiar/frequent words, drastically deteriorated at visit 2. Thus, the pattern of aphasia at visit 2 changed from logopenic progressive aphasia to progressive Wernicke's aphasia, characterized by poor repetition and word comprehension (6). Case 8 also showed extensive and severe deficits in cognitive domains other than language.

### 3.4. Lesion distribution on SPECT

**Figure 2:**
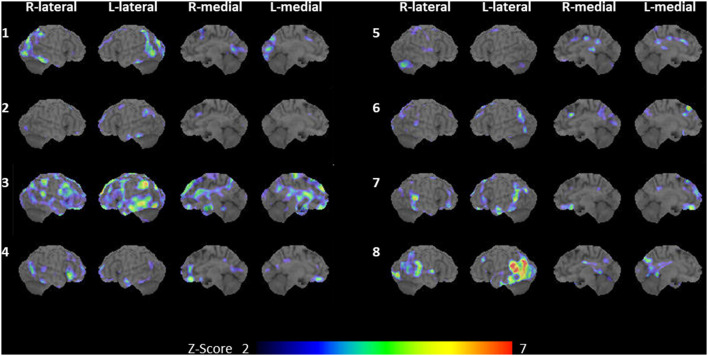
Brain SPECT. IMP-SPECT analyzed with 3D stereotactic surface projections revealed patterns of significant hypoperfusion in each of the eight cases with an aphasic MCI-LB. Case numbers are shown to the left of each set of images. Cases 1–5: progressive anomic aphasia; cases 6–8: logopenic progressive aphasia. L, left; R, right; aphasic MCI-LB, aphasic mild cognitive impairment with Lewy bodies; IMP-SPECT, *N*-isopropyl-p-[^123^I] iodoamphetamine single-photon emission computed tomography.

The IMP-SPECT-3D SSP analysis of each patient is shown in Figure 2. The degree of hypoperfusion varied between patients; when present, it most often involved the lateral or medial occipital lobe (seven patients: cases 1–4, and 6–8), revealing potential biomarkers of MCI-LB (2) and supportive biomarkers of DLB (3). In addition, there was no hypoperfusion in the precuneus and posterior cingulate cortex in two out of the seven patients (cases 2 and 7), indicating the cingulate island sign, which is one of the supportive biomarkers of DLB (3). In contrast, hypoperfusion in the remaining six patients was noted in the precuneus or posterior cingulate cortex, indicating a surrogate marker of early Alzheimer's disease (44). The left temporal and parietal lobes were involved in all but one patient; hypoperfusion in the right temporoparietal lobe was present in case 5. Compared with the five patients (cases 1–5) with progressive anomic aphasia, the three patients (cases 6–8) with logopenic progressive aphasia had extensive hypoperfusion in the left temporoparietal cortices, including Wernicke's area.

### 3.5. Follow-up data of three patients with medication

Three patients (cases 1, 4, and 8) underwent cholinesterase inhibitor therapy with donepezil, and all patients showed improvement in general cognitive functions, including language function. Case 8 had rapid temporary improvement in language and other cognitive functions, based on bedside examinations by a behavioral neurologist and speech and language therapist. The pattern of aphasia in case 8 improved from progressive Wernicke's aphasia at visit 2 to a relatively similar pattern of logopenic progressive aphasia observed at visit 1. However, the effect lasted for only 1 week.

The remaining two patients (cases 1 and 4) also underwent language and neuropsychological assessments, and we compared their language and cognitive impairments at baseline with their performance at follow-up sessions after donepezil treatment. Follow-up started 3 months after the donepezil dosage was increased to 5 mg/day (16); we observed improvements in the patients' total WAB-Aphasia quotient scores and TLPA-naming tasks, and in the total MMSE and ACE-R scores (Table 3).

**Table 3:** Follow-up data of two patients with medication.

	**Case 1**		**Case 4**			**Normative data, mean (SD)**
			**Visit 1**	**Visit 2**		
	**Baseline**	**Follow-up**		**Baseline**	**Follow-up**	
**Demographic characteristics**						
MMSE total score (30)	28	30	29	28	29	28.6 (1.4)
ACE–R total score (100)	79	98	83	72	81	91.1 (8.4)
ADAS recall (30)	9	10	17	10	11	21.5 (2.8)
ADAS recognition (36)	31	36	35	31	35	31.3 (3.9)
Digit span	F5B3	F6B3	F6B4	F7B5	F5B4	
Spatial span	F4B3	F4B4	F5B5	F6B5	F5B4	
Noise pareidolias (32)	0	0	DNT	4	0	0.2 (0.4)
**WAB**						
Aphasia quotient (100)	93.6	95.2	85.4	82.8	90.2	97.7 (3.0)
Fluency (10)	9	9	8	8	9	10.0 (0)
Information content (10)	10	10	8	8	9	9.7 (0.6)
Auditory comprehension (10)	8.9	9.6	9.1	9.5	9.8	9.8 (0.1)
Repetition (10)	10	10	9.9	8.4	9.4	9.9 (0.3)
Naming (10)	8.9	9	7.7	7.5	7.9	9.5 (0.6)
Praxis (60)	60	60	60	60	60	59.8 (0.7)
**TLPA**						
Naming (200)	178	191	119	101	147	193.4 (5.4)
Comprehension (200)	200	200	193	155	193	199.4 (1.0)
Severity of word comprehension impairment	–	–	^*^	^***^	^*^	
**Types of aphasia**						
	A	A	A	T	A	

A, progressive anomic aphasia; ACE-R, Addenbrooke's Cognitive Examination-Revised; ADAS, Alzheimer's Disease Assessment Scale; B, backward; DNT, did not test; F, forward; MMSE, Mini–Mental State Examination; SD, standard deviation; T, progressive transcortical sensory aphasia; TLPA, Test of Lexical Processing in Aphasia; WAB, Western Aphasia Battery. The maximum score is noted in each row header. Severity of word comprehension impairment: ^*^, minimal; ^**^, mild; ^***^, severe.

## 4. Discussion

The present study demonstrated the clinical and imaging features of eight patients with aphasic MCI, including PPA, in prodromal DLB. Five patients (cases 1–5) were diagnosed with progressive anomic aphasia, and three patients (cases 6–8) with logopenic progressive aphasia. These characteristics of language impairment were similar to those seen in Alzheimer's disease. In our aphasic MCI cohort, the clinical diagnosis of probable MCI-LB accounted for more than 30% of cases; therefore, the presence of language impairment in prodromal state of DLB was not uncommon. Moreover, three patients who received cholinesterase inhibitors showed improvement in general cognitive function, including language function. These findings provide further insight into the clinical spectrum of prodromal DLB.

In our cohort of 26 patients with aphasic MCI, eight patients (30.8%) met the criteria for probable MCI-LB. In addition, in our cohort of 20 patients with PPA, two patients (10%) met the criteria for probable MCI-LB. The prevalence of prodromal DLB in patients with aphasic MCI, including PPA, was higher than expected (10), because language function is relatively preserved in patients with typical DLB or MCI-LB compared with attention, executive function, and visual perception. Furthermore, in our cohort of seven patients with logopenic progressive aphasia, three patients (42.9%) met the criteria for probable MCI-LB. Similarly, in our cohort of 12 patients with progressive anomic aphasia, considered a prodromal state of logopenic progressive aphasia (6), 5 patients (41.7%) met the criteria for probable MCI-LB. Consistent with our findings, a recent clinicopathological study (8) demonstrated that seven (41.2%) of 17 prospectively recruited patients with lvPPA were positive for Lewy body pathology. In our cohort, there was no prodromal DLB in patients with the typical features of nfvPPA or svPPA, but Lewy body disease may be considered as a pathology of logopenic progressive aphasia and unclassified progressive fluent aphasia.

The features of progressive anomic aphasia and logopenic progressive aphasia are known to be similar to the progressive aphasia observed in Alzheimer's disease (6, 20, 21). Previous studies have shown that the most common feature of language impairments in early-stage Alzheimer's disease was progressive anomic aphasia without impaired repetition and word comprehension, which progressed to logopenic progressive aphasia with impaired repetition or progressive TCSA with impaired word comprehension. Both logopenic progressive aphasia and progressive TCSA in late-stage Alzheimer's disease eventually advanced to progressive Wernicke's aphasia, with impaired repetition and word comprehension (6, 45). Consistent with previous studies of language impairments in Alzheimer's disease, the prevalence of progressive anomic aphasia was also the highest in our cohort of patients with aphasic MCI-LB. In addition, in three patients who were followed up in our study, progressive anomic aphasia (cases 2 and 4) progressed to logopenic progressive aphasia or progressive TCSA, and logopenic progressive aphasia (case 8) progressed to progressive Wernicke's aphasia. Moreover, all patients showed hypoperfusion of the temporal and parietal lobes on SPECT. In progressive anomic aphasia (cases 1–5), the affected area varied across cases, consistent with our previous study on anomic aphasia in Alzheimer's disease (6). The temporoparietal lobe, including Wernicke's area, was involved in logopenic progressive aphasia (cases 6–8), and these regions are known to be associated with logopenic progressive aphasia in Alzheimer's disease (5). Therefore, the clinical and imaging features of language impairments in prodromal DLB may resemble those observed in Alzheimer's disease.

Mixed pathologies, such as amyloid pathology in neocortical areas, have been considered the underlying cause of cognitive and language impairments (11, 46). Co-pathology with amyloid deposits could be considered in six of our eight patients (75%), as the 3D SSP analysis on IMP-SPECT revealed reduced blood flow in the posterior cingulate gyrus and/or precuneus; this is a surrogate marker of early Alzheimer's disease (44). However, although a majority (55–82%) of typical DLB patients have amyloid pathology in the posterior neocortical areas including temporoparietal cortices (46, 47), language function is usually relatively preserved. This suggests that amyloid pathology cannot always be explained as the primary cause of language impairment in DLB. Further, Buciuc et al. (8) reported a relationship between the cortical distribution of α-synuclein pathology and language impairment in prodromal DLB. In addition, cholinergic insufficiency is one of the mechanisms underlying general cognitive impairment in DLB and MCI-LB (2, 3). The efficacy of cholinesterase inhibitors was demonstrated in our study. Although we did not compare the efficacy of donepezil between prodromal DLB and Alzheimer's disease, cholinergic insufficiency may have a greater effect on language impairment in prodromal DLB (15, 16). The underlying cause of language impairment in prodromal DLB, including the effects of α-synuclein and amyloid deposits or cholinergic dysfunction, are unclear in the present study and need to be determined.

Recent research findings have suggested that prodromal DLB (2) has a variety of clinical symptoms such as delirium, psychiatric symptoms, olfactory dysfunction, and dysautonomia, with relatively preserved language function. However, recent studies have demonstrated that some patients (8, 9, 11, 12, 14–16) have predominantly impaired language function in MCI-LB. Moreover, two of our patients (cases 4 and 8) did not show any DLB-related clinical features in the early stages; thus they also met the core criteria for PPA (5). Therefore, aphasic MCI including PPA may be considered as one of the clinical presentations in the prodromal states of DLB (16), and our results provide insight into the spectrum of prodromal DLB.

The efficacy of cholinesterase inhibitors in treating general cognitive impairments and visual hallucinations has been demonstrated in various studies, as cholinergic insufficiency is a well-known mechanism of these clinical symptoms (2, 3, 48, 49). Recent case studies have suggested that donepezil may be effective in treating progressive anomic aphasia (16) and logopenic progressive aphasia (15) in prodromal DLB. Consistent with these previous studies, donepezil was effective in improving general cognitive and language functions in three of our patients, despite their not having received speech and language therapy for progressive aphasia. Our findings may thus contribute to the development of medication for progressive aphasia.

The present study has several limitations. First, our study design was a retrospective case series. As the cohort focused primarily on progressive aphasia, the assessments of DLB-related clinical features were inadequate compared with the assessments of language features. In addition, regarding medication for three patients, we did not investigate the efficacy of donepezil on progressive aphasia in prodromal DLB in a randomized clinical trial. Improvements in general cognitive and language functions after administering donepezil might also reflect fluctuating cognition in prodromal DLB, although qualitative examinations confirmed that none of the patients had severe fluctuating cognition. Second, neuropathological examinations were not performed. Specifically, the influence of Alzheimer's disease on progressive aphasia cannot be excluded, and hence this is the most significant limitation of our study. Therefore, further investigations are needed to determine the etiology of aphasia in prodromal DLB.

In conclusion, the clinical and imaging features of language impairment in MCI-LB resemble those observed in Alzheimer's disease. Progressive fluent aphasia, such as progressive anomic aphasia and logopenic progressive aphasia, is one of the clinical presentations in prodromal state of DLB. The findings of this study provide further insight into the clinical spectrum of prodromal DLB and can contribute to developing medication for progressive aphasia caused by cholinergic insufficiency.

## Data availability statement

The original contributions presented in the study are included in the article/supplementary material, further inquiries can be directed to the corresponding author.

## Ethics statement

The studies involving human participants were reviewed and approved by the Ethics Committee of Nippon Life Hospital. The patients/participants provided their written informed consent to participate in this study. Written informed consent was obtained from the individual(s) for the publication of any potentially identifiable images or data included in this article.

## Author contributions

HW and EM contributed to the concept and design of the study. HW contributed to drafting the manuscript and preparing the figures and tables. EM and MI revised the manuscript. All authors were instrumental in data acquisition and analysis. All authors contributed to the article and approved the submitted version.
